# Ginnalin A and hamamelitannin: the unique gallotannins with promising anti-carcinogenic potential

**DOI:** 10.37349/etat.2023.00129

**Published:** 2023-04-21

**Authors:** Rippin, Vikas Beniwal, Ajay Sharma, Bikram Jit Singh, Seema Ramniwas, Katrin Sak, Satish Kumar, Anil K. Sharma

**Affiliations:** 1 Department of Biotechnology, Maharishi Markandeshar Engineering College, Maharishi Markandeshwar (Deemed to be University), Haryana 133207, India; 2 Department of Microbiology, Central University of Haryana, Haryana 123029, India; 3 Department of Chemistry, Career Point University, Hamirpur 176041, Himachal Pradesh, India; 4 Department of Mechanical Engineering, M.M. Engineering College, Maharishi Markandeshwar (Deemed to be University), Haryana 133207, India; 5 University Centre for Research and Development, University Institute of Biotechnology Chandigarh University, Mohali 140413, India; 6 Non-government Organization, Praeventio, Tartu 50407, Estonia; 7 College of Horticulture and Forestry, Thunag, Dr. Y. S. Parmar University of Horticulture and Forestry, Solan 173230, India; University of East Anglia, UK

**Keywords:** Tannins, gallotannins, ginnalin A, acertannin, anti-carcinogen, hamamelitannin

## Abstract

Tannins are secondary metabolites that belong to the family of polyphenolic compounds and have gained a huge interest among researchers due to their versatile therapeutic potential. After lignin, these are the second most abundant polyphenols found in almost every plant part like stem, bark, fruit, seed, leaves, etc. Depending upon their structural composition, these polyphenols can be divided into two distinct groups, namely condensed tannins and hydrolysable tannins. Hydrolysable tannins can be further divided into two types: gallotannins and ellagitannins. Gallotannins are formed by the esterification of *D*-glucose hydroxyl groups with gallic acid. The gallolyl moieties are bound by a depside bond. The current review focuses mainly on the anti-carcinogenic potential of recently discovered gallotannins, ginnalin A, and hamamelitannin (HAM). Both of these gallotannins possess two galloyl moieties linked to a core monosaccharide having anti-oxidant, anti-inflammatory, and anti-carcinogenic abilities. Ginnalin A is found in plants of the genus *Acer* whereas HAM is present in witch hazel plants. The biosynthetic pathway of ginnalin A along with the mechanism of the anti-cancer therapeutic potential of ginnalin A and HAM has been discussed. This review will certainly help researchers to work further on the chemo-therapeutic abilities of these two unique gallotannins.

## Introduction

**Figure 1.**
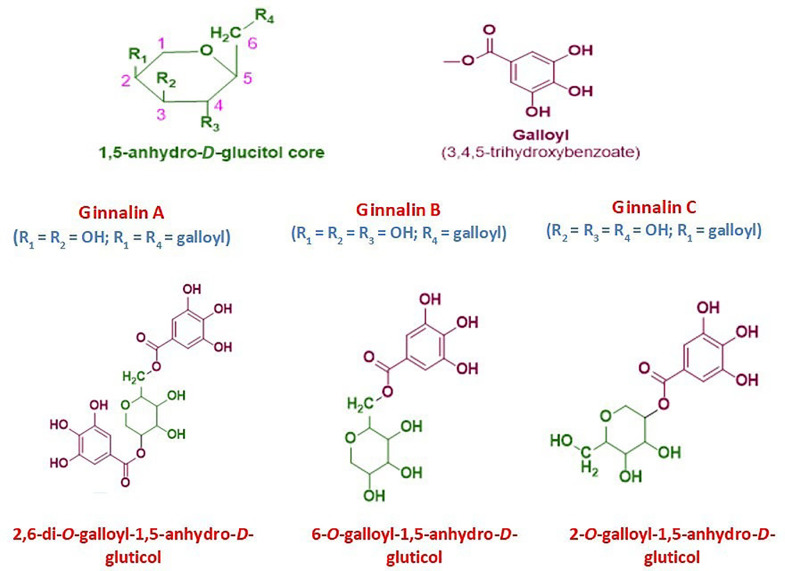
The general chemical structure of 1,5-anhydro-*D*-glucitol core moiety with numbering and substituents position and galloyl group. The chemical structure of ginnalin A, ginnalin B, and ginnalin C has been shown along with their systematic names and substitution pattern of hydroxyl groups and galloyl group on 1,5-anhydro-*D*-glucitol core

Tannins may be found in nature in a wide range of plants parts. Tannins can cross-link with proteins and other macromolecules because they have several hydroxyl or other functional groups on their skeleton. Proanthocyanidins and hydrolysable tannins are more or less how tannins are classified and fulfilled for therapeutic purposes and health benefits [1]. Hydrolysable tannins are further grouped into gallotannins and ellagitannins [2]. Ginnalins are natural compounds based on glucitol-core (1,5-anhydro-*D*-glucitol) containing gallotannins and belong to the phenolic class of plant metabolites. These phenolic compounds are also known as gallic acid metabolites in which the galloyl groups attached to different positions of glucitol-core moiety. These are generally known as ginnalin A, ginnalin B, and ginnalin C which are mainly characterized on the basis of the number of galloyl (3,4,5-trihydroxybenzoate) groups and their position of attachment to 1,5-anhydro-*D*-glucitol core moiety. This core moiety is a six-carbon monosaccharide with a pyranoid ring structure. Ginnalins B and C are known as monogalloyl gallotannins while the ginnalin A is di-galloyl gallotannin compound (see Figure 1). The galloyl group attached at 6-position and 2-position of 1,5-anhydro-*D*-glucitol in ginnalins B and C, respectively while in ginnalin A, the two galloyl groups attached at 6-position and 2-position of 1,5-anhydro-*D*-glucitol [3–5].

This unique metabolite, ginnalin A can be derived from leaves of *Acer ginnala* and *Acer tataricum* subsp. *ginnala* [6–8]. It has also been reported to be found in *Acer ginnala* Maxim. leaves, twigs, and bark [9]. *Acer rubrum* L. buds also contain an ample amount of ginnalin A [10]. It can also be recovered from the leaves and twigs of *Acer okamotoanum* Nakai [11]. Many authors have used various methods to extract this compound. After extraction of leaves of *Acer ginnala*, thrice with methanol, vacuum drying, the addition of water, and filtration, four fractions were obtained from sephadex column chromatography. The 3rd fraction after crystallisation yielded 80 mg of ginnalin A per g fresh weight [6]. Hot water extract from *Acer rubrum* buds has exclusively been studied for anti-oxidant potential [12].

**Figure 2.**
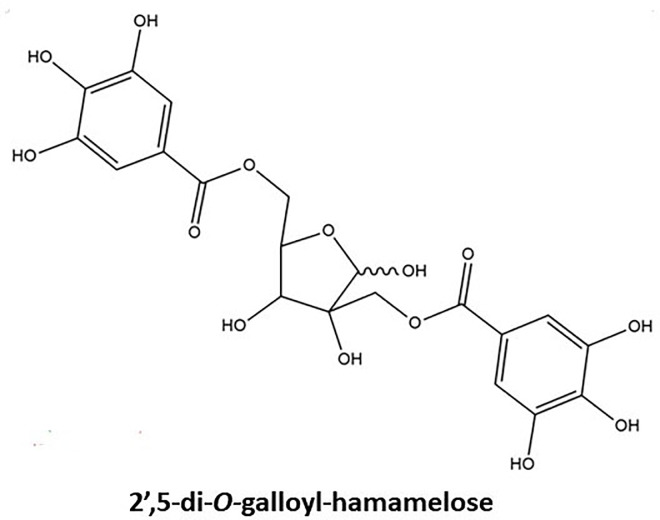
Basic chemical structure of HAM. Two galloyl moieties are esterified to hamamelofuranose core moiety

Hamamelitannin (HAM) also known as 2’,5-di-*O*-galloyl-*D*-hamamelose belongs to gallotannins. This secondary metabolite is found in *Hamamelis virginiana* L. (witch hazel) plants especially barks [13]. The structure of HAM has been illustrated in Figure 2. This gallotannins possesses two galloyl moieties which are bound to a central core molecule which is a furanose. HAM has also been found to be present in leaves and twigs of the witch hazel plant. It was isolated from the plant parts by using a separation system that included a C18 reversed-phase column, a gradient elution system utilizing methanol/water and orthophosphoric acid, and a photodiode array detector, respectively [14]. Both ginnalin A and HAM contain two galloyl groups linked to a core sugar. Due to this structural similarity both of these compounds stand apart from other gallotannins and have been discussed ahead in this review. The main focus is on the structure and the biosynthetic pathway of the gallotannins, ginnalin A in particular. Both the gallotannins are known to possess promising therapeutic potential with HAM being an established quorum-sensing inhibitor. Both tannins require to be further investigated by researchers for their anti-cancer abilities as both may be helpful in the chemotherapeutic treatment of cancer.

## Postulated biosynthesis pathway of ginnalin A

**Figure 3.**
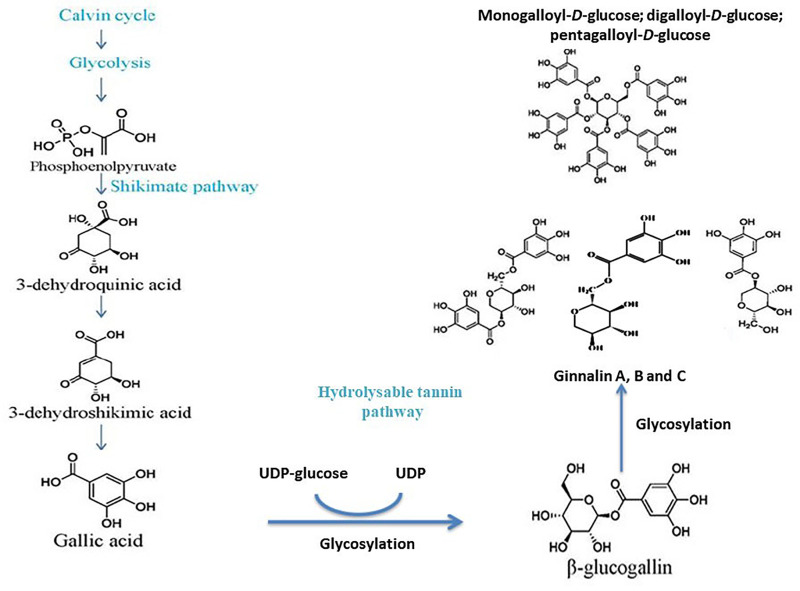
Postulated biosynthesis of monogalloyl-*D*-glucose, digalloyl-*D*-glucose, pentagalloyl-*D*-glucose to form ginnalin A, B, and C. Both ginnalin A and HAM have striking similarities in structure as both have a core monosaccharide moiety linked with two galloyl molecules. So, it is anticipated that both will probably follow the same pathway

The brief outline of galloylglucose biosynthesis pathway involves the formation of gallic acid through the shikimate pathway and β-glucogallin through the enzymatic action of uridine diphosphate (UDP)-glucose as shown in Figure 3.

The calvin cycle and glycolysis result in the formation of phosphoenolpyruvate. The shikimate pathway of gallic acid formation involves the conversion 3-dehydroshikimic acid into gallic acid followed by the conversion of phosphoenolpyruvate into 3-dehydroquininc acid and further into the 3-dehydroshikimic acid. The glycosylation of gallic acid takes place further into β-glucogallin with UDP-glucose and the specific intermediate of hydrolysable tannins. The glucose exchange reactions as shown by the β-glucogallin (specific galloyl donor) into either mono-/di-/tri-/tetra-galloyl-*D*-glucose or penta-galloyl-*D*-glucose by the action of β-glucogallin dependent galloyl transferase [13, 14].

## Therapeutic potential of ginnalin A and HAM

**Figure 4.**
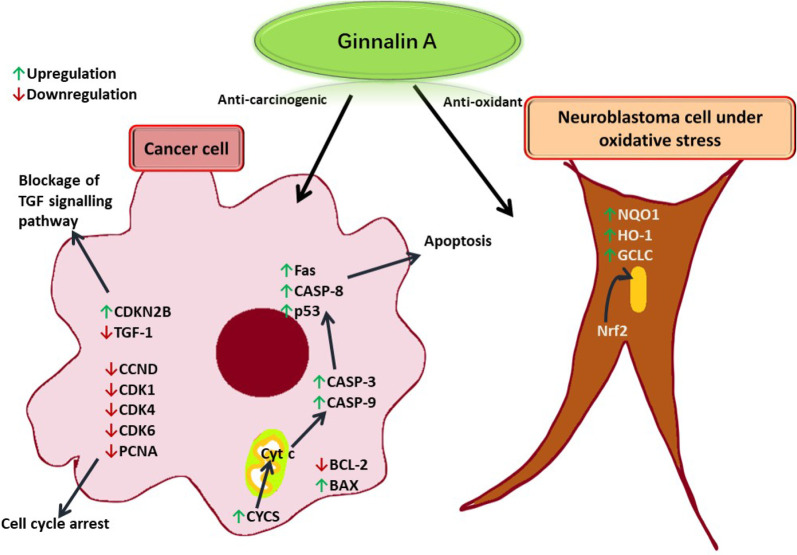
Ginnalin A induces apoptosis in a cancer cell by enhancing the release of Cyt c from the mitochondria to produce apoptotic effector proteinases. TGF: tumor growth factor; CDKN2B: cyclin-dependent kinase inhibitor 2B; CCND: cyclin D1 protein gene; CDK1: cyclin-dependent kinase 1; PCNA: proliferating cell nuclear antigen; Fas: FS-7-associated surface; CASP-8: caspase-8; p53: tumor protein 53; BCL-2: B cell lymphoma-2; BAX: BCL-2-associated X protein; Cyt c: cytochrome c; CYCS: Cyt c synthetase

Ginnalin A isolated from the methanolic extract of *Acer ginnala* leaves showed a remarkable antioxidant effect *in vitro*. Its 1,1-diphenyl-2-picrylhydrazyl (DPPH) radical scavenging half-maximal drug inhibitory concentration (IC_50_) and nitroblue tetrazolium (NBT)/superoxide scavenging IC_50_ values were reported to be significantly low, respectively [6]. Hot water extract from *Acer rubrum* buds showed appropriate anti-radical activity and neutrophil inhibiting potential as it was found to contain ginnalin A besides other polyphenols [12]. In another study, ginnalin A acted as an indirect reactive oxygen species (ROS) scavenger and activated the nuclear factor erythroid 2-related factor 2 (Nrf2) pathway in SH-SY5Y cells (neuroblastoma cell line) exposed to 6-hydroxydopamine (6-OHDA). After pre-treatment with ginnalin A, Nrf2 dissociated from the Kelch-like epichlorohydrin-associated protein 1 (Keap1)-Nrf2 complex and translocated into the nucleus. There was a remarkable increase in nicotinamide adenine dinucleotide phosphate [NAD(P)H] quinone oxidoreductase-1 (NQO1), heme oxygenase-1 (HO-1), and the glutamate-cysteine ligase catalytic (GCLC) subunit and elevated glutathione concentration (see Figure 4) [15].

The cell cycle is also arrested due to the downregulation of CDK cyclins. In a neuroblastoma cell under oxidative stress, ginnalin A promoted the translocation of Nrf2 to the nucleus thereby releasing enzymes that quenched free radicals.

Analyzing the human blood neutrophil-mediated inflammation, it was deduced that ginnalin A elevated Fas-associated protein with death domain (FADD), phospho-Rad17 antibody, second mitochondrial-derived activators of CASP (SMAC)/direct inhibitor of apoptosis protein-binding protein with low pI (Diablo), and Cyt c while lowering the anti-apoptotic protein catalase in neutrophils, which led to the induction of apoptosis [10]. The exposure of human keratinocytes to ginnalin A led to the inhibition of apoptosis by upregulating BH3-interacting domain death agonist (Bid), BCL-2, and B cell lymphoma-extra large (BCL-xL) and incrementing survivin levels. A remarkable decrease was observed in BAX, Cyt c, CASP-8, CASP-9, CASP-3, and p53 levels. Ginnalin A prevented the tumor necrosis factor (TNF)-related apoptosis-inducing ligand (TRAIL)-induced formation of reactive oxygen/nitrogen species, apoptosis-related protein activation, and cell death [16]. While studying the hydrogen peroxide-induced oxidative stress in keratinocyte human immortalized keratinocytes (HaCaT) cells, it was observed that the treatment with ginnalin A reduced cell toxicity and increased cell viability [7]. The mechanism of action of ginnalin A has been depicted in Figure 3. The TRAIL causes inflammation and release of reactive oxygen and nitrogen species in human keratinocytes leading to apoptosis and atopic dermatitis.

While investigating the combined effect of ginnalin A and SB203580 [a p38 mitogen-activated protein kinase (MAPK) inhibitor] on Hep-3B, a hepatocellular carcinoma cell, it was observed that the treatment upregulated the expression of apoptotic protein gene *BAX* (responsible for the release of Cyt c from mitochondria to cytosol), whereas expression of Cyt c blocking protein gene *BCL-2* was downregulated. The expression of Cyt c encoding protein CYCS was also enhanced. The release of Cyt c is responsible for the activation of CASP-3 and CASP-9 effector proteinases, which further leads to apoptosis. It was observed that the expression of these proteinases as well as CASP-8, and p53 was enhanced. The expression of CCND1, CDK1, and CDK4 was adversely affected, which led to the arrest of the G1 phase of the cell cycle [17]. When DLD-1, a colorectal cell line, was exposed to dark maple syrup, the cell cycle was arrested, and PCNA expression was downregulated. Further research revealed that CDK4/6 kinase expression was suppressed. TGF-1 was then downregulated as a result, and CDKN2B expression was subsequently increased. Consequently, the TGF signalling pathway was blocked [18]. Ginnalin A was effective against hepatocellular carcinoma as it showed a distinguished *in vitro* apoptotic effect on Hep-3B human hepatocellular carcinoma cell line. There was a significant upregulation observed in the expression of *CASP-3*, *CASP-8*, *CASP-9*, *CYCS*, and *p53* genes [19]. In another study, on human breast cancer cell lines, it was observed that there was a marked increase in *CASP-3*, *CASP-8*, *CASP-9*, *CYCS*, *FAS*, and *p53* genes expression in MDA-MB-231 cell line. In the case of MCF-7, a breast cancer cell line, with ginnalin A dose, *CASP-9*, and *p53* gene expression was upregulated while *BCL-2* gene expression was significantly downregulated [20]. In another study, the first-ever mechanism of action of ginnalin A against the human neuroblastoma cell line SH-SY5Y and the mouse neuroblastoma cell line N18TG2 proliferation was revealed. Ginnalin A’s IC_50_ values against the cell lines ranged from 70 g/mL to 150 g/mL, which were significant enough [9]. In yet another experiment on the murine melanoma B16F10 cell line, it was inferred that ginnalin A not only suppressed the expression of tyrosinase and melanogenic gene but also alleviated oxidative stress [7]. It also showed some cytotoxicity against L1210, HL-60, K562, and B16F10 cancer cell lines *in vitro* by 3-(4,5-dimethylthiazol-2-yl)-2,5-diphenyltetrazolium bromide (MTT) assay [11]. Ginnalin A inhibited the proliferation of HCT-116 and MCF-7 cell lines and it also obstructed the S and G2/M-phases of the cell cycle by down-regulating the levels of cyclin A and cyclin D1 [21]. Ginnalin A inhibited the proliferation of HCT-116, HT-29, and Caco-2 cell lines by blocking the S-phase of the cell cycle [22]. Out of three colon cancer cell lines HCT116, SW480, and SW620, ginnalin A was most effective against SW480 [23]. It not only inhibited the formation of colonies but also caused hindrance of the S-phase of the cell cycle, thus adversely affecting the proliferation. Nuclear translocation of Nrf2 was observed along with the enhancement of *Nrf2*, *HO-1*, *p62*, and *NQO1* genes [24]. Down-regulation of Keap1 was also observed which led to the initiation of Nrf2 signalling pathway [8].

Similarly, HAM was also reported to be an anticancer, anti-inflammatory, antioxidant agent as well as a quorum sensing inhibitor as established in earlier studies in the literature [25–31]. It was observed in a study that HAM possessed anti-colon cancer activities as it showed lower IC_50_ values against various colorectal cell lines in MTT assay. The cell lines were SW620, DLD-1, HT29, HCT8, and HCT116 and the standard used was doxorubicin [27]. In another set of experiments, HAM was found to be active against chemokine (C-C motif) ligand 26 (CCL26), K10, and cell proliferation and hence hindered the interleukin-4 (IL-4) inflammatory pathway. Thus, it was found to be effective against the keratinocyte inflammatory cascade [28]. In yet another set of experiments, the murine skin fibroblasts were exposed to ultraviolet (UV)-B (UVB) radiation. It was inferred that HAM had potent activity against the ROS and hence protected the fibroblasts. HAM was found to be more effective against superoxide anions than gallic acid and was proved to be the top-runner for protecting the murine dermal fibroblasts from UV-induced damage [29]. HAM also possessed the ability to depolymerize hyaluronic acid along with radical scavenging capacity [30]. HAM has also shown a protective effect against cell death of endothelial cells, EAhy926. The cell death was reportedly induced by TNF-α [31]. In yet another set of experiments, it was observed that HAM was able to protect the Hep-G2 cells from DNA damage induced by procarcinogen benzo(a)pyrene. It was further inferred that the gallotannin was still able to protect the cells from DNA damage when they were exposed to the mutagen (±)-anti-benzo(a)pyrene-7,8-dihydrodiol-9,10-epoxide. The mutagen was incapacitated by the formation of phenol-mutagen-adduct [32]. In another study, HAM has been reported to be a quite effective scavenger of superoxide and hydroxyl radicals and in protection against DNA damage [33].

Keeping in view of the above facts, it is anticipated that the anti-cancer activity of these gallotannins has been attributed to the presence of a phenolic group. It was also evident from previous studies by Sánchez-Tena et al. [26] in *Hamamelis virginiana* bark was reported to have a protective action against colon cancer, as these gallotanins are enriched with condensed and hydrolyzable tannins. HAM was reported not only to induce apoptosis, necrosis, and cell cycle arrest in S-phase but also to reduce the viability of the tumor [26].

## Conclusions

Ginnalin A and HAM are unique secondary metabolites that belong to gallotannins family. Ginnnalin A is present in *Acer* plant species whereas HAM comes from the witch hazel plant. Both of these compounds possess two galloyl groups in their structure. In ginnalin A, these are esterified to a glucose moiety whereas in HAM they are bound through an ester bond to hamamelofuranose moiety, respectively. Both gallotannins portray excellent anti-carcinogenic potential. The therapeutic potential of these molecules can be explored further as both of these are perfect candidates for becoming great nutraceuticals. However, a large cohort of clinical studies is required in order to further strengthen and establish the anti-carcinogenic potential of ginnalin A and HAM. The biosynthesis of these gallotannins is yet to be deciphered.

## Abbreviations

BAX: B cell lymphoma-2-associated X protein
BCL-2: B cell lymphoma-2
CASP-8: caspase-8
CDK1: cyclin-dependent kinase 1
CYCS: cytochrome c synthetase
Cyt c: cytochrome c
HAM: hamamelitannin
HO-1: heme oxygenase-1
IC_50_: half-maximal drug inhibitory concentration
NQO1: nicotinamide adenine dinucleotide phosphate quinone oxidoreductase-1
Nrf2: nuclear factor erythroid 2-related factor 2
p53: tumor protein 53
TGF: tumor growth factor
UDP: uridine diphosphate

## Declarations

### Copyright

© The Author(s) 2023.
